# The effect of mannitol addition to hydration on acute kidney injury event after high dose cisplatin chemotherapy: an ambispective cohort study

**DOI:** 10.1186/s12885-022-09456-w

**Published:** 2022-04-12

**Authors:** Andhika Rachman, Syahidatul Wafa, Pringgodigdo Nugroho, Sukamto Koesnoe

**Affiliations:** 1 Department of Internal Medicine, Division of Hematology and Medical Oncology, Dr. Cipto Mangunkusumo General Hospital – Faculty of Medicine, Universitas Indonesia, Jl. Pangeran Diponegoro No.71, RW.5, Kec. Senen, Central Jakarta, Jakarta, 10430 Indonesia; 2 Department of Internal Medicine, Dr. Cipto Mangunkusumo General Hospital - Faculty of Medicine, Universitas Indonesia, Jakarta, Indonesia; 3 Department of Internal Medicine, Division of Nephrology and Hypertension, Dr. Cipto Mangunkusumo General Hospital – Faculty of Medicine, Universitas Indonesia, Jakarta, Indonesia; 4 Department of Internal Medicine, Division of Allergy and Immunology, Dr. Cipto Mangunkusumo General Hospital – Faculty of Medicine, Universitas Indonesia, Jakarta, Indonesia

**Keywords:** High dose cisplatin, Mannitol, *Acute kidney injury*

## Abstract

**Background:**

Saline hydration with addition of mannitol have commonly been the strategy to avoid cisplatin induced acute kidney injury (AKI). While the initial reports demonstrated that mannitol diuresis decreased cisplatin induced renal injury, others have shown renal injury to be worsened.

**Objective:**

To compare the risk of AKI in cancer patients receiving high dose cisplatin with and without addition of mannitol.

**Method:**

This was an ambispective cohort study based on consecutive sampling at Cipto Mangunkusumo General Hospital (CMGH) and Mochtar Riady Comprehensive Cancer Centre (MRCCC) Siloam Hospitals. The data was obtained from September 2017 to February 2018. The choice of mannitol administration based on attending physician clinical judgement. The primary outcome was increase of serum creatinine more than 0.3 mg/dL or 1.5 times from baseline. Analysis was done by using univariate, bivariate and multivariate logistic regression to obtain crude risk ratio and adjusted risk ratio of cisplatin induced AKI probability caused by mannitol addition on top of usual saline hydration protocol.

**Result:**

Data from 110 patients (57.3% male) with a median age of 44.5 years (range 19 to 60 years) were collected; 63 received saline with the addition of mannitol and 47 received saline only. Incidence of AKI were higher in mannitol vs saline only group. Bivariate analysis showed higher probability of post chemotherapy AKI in mannitol group, however it was statistically insignificant (RR 2.168; 95% CI 0.839–5.6; *p* = 0.094). On multivariate analysis the age adjusted RR was 2.852 (95% CI 0.68–11.96; *p* = 0.152).

**Conclusion:**

The addition of mannitol to hydration did not reduce the risk of cisplatin induced AKI as compared with saline hydration only. It was also found that risk for acute kidney injury were higher in population ≥ 40 years old.

## Introduction

Cisplatin is a platinum-based chemotherapy agent that currently used as front-line therapy in the treatment of various solid organ cancers, including head and neck, lung, testis, ovary, breast, bladder cancer and sarcoma [1–7]. The therapeutic effects of cisplatin are significantly improved by dose escalation. However, high dose therapy of cisplatin is limited by significant side effects, such as nephrotoxicity, neurotoxicity, ototoxicity and emetogenicity [8–10]. In particular, renal toxicity occurs in 20%-40% of patients in a dose dependent manner, therefore limits the amount of drug that can be administered [11–13].

Cisplatin injures mitochondrial DNA, mostly at the third segment of proximal tubular cells, result in renal mitochondrial dysfunction. This injury led to a decline in adenosine triphosphate production. Reduced Na–K-ATPase activity leads to altered intracellular-extracellular sodium gradient, which inhibits normal sodium reabsorption. The reduction in renal blood flow (RBF) and glomerular filtration rate (GFR) is due to increased renal vascular resistance, activated by tubuloglomerular feedback from increased sodium delivery to the macula densa in the early distal tubule. Additionally, cisplatin could directly induce necrosis and apoptosis of renal tubular cells, resulting an inflammation and oxidative stress that worsen the renal injury [10, 11, 14, 15]. The decrease in the glomerular filtration rate causes an increase in serum creatinine within 6 to 7 days and tends to remain elevated for 3–4 weeks after cisplatin administration [16–19].

Although renal impairment is transient and reversible, 43% of patients with AKI went on to develop an irreversible renal failure [20]. Nephrotoxicity was the main reason of discontinuation of chemotherapy and poor survival of patients [12, 13, 20–23]. The main protective measures currently employed in clinical practice are based on avoiding the excessive exposure of cisplatin to the kidneys, basically by hydration and diuretics, such as mannitol, since it was filtered but not reabsorbed by the kidneys, mannitol remains in the renal tubules and causes an increase in the delivery of sodium to the distal tubules and a continued osmotic diuresis. This results in a “flushing” effect in the renal tubules thus reducing cisplatin contact time with tubular cells, preventing it from evolving into toxic compounds that damage the kidneys and increase the elimination of cisplatin in the urine [24–26]. On the other hand, potential nephrotoxicity of mannitol raised clinician’s concerns. When administered to patients with normal kidney function, mannitol may cause hypokalemia due to increased flow rates in the aldosterone-responsive distal nephron which leads to increased potassium loss. In contrast, given to patients with preexisting kidney failure, mannitol is found to be retained in circulation and may cause extracellular fluid volume expansion, hyponatremia, metabolic acidosis, and hyperkalemia [27, 28]. Moreover, mannitol may induce extensive isometric renal proximal tubular vacuolization and intense afferent arteriolar constriction resulting in AKI [29, 30].

Meta-analysis from Bo Yang et al. in adult patients at increased risk of AKI revealed that intravascular administration of mannitol does not convey additional benefit beyond adequate hydration [31]. The role of mannitol as prevention of cisplatin-associated AKI has been investigated in some trials, but the result was conflicting whether nephroprotective or nephrotoxic so no definite recommendation regarding the nephroprotective effect of mannitol has emerged.

The non-comparative study by Hayes (1977)[16] was the first to state the advantage of hydration plus mannitol to reduce the risk of cisplatin nephrotoxicity. Then, the prospective phase II trial by Al-Sarraf (1982) [32] supported the results of the Hayes study (incidence of nephrotoxicity in mannitol plus hydration was 15% vs. 30% in hydration alone), unfortunately this result was not statistically validated and in the next cycle of cisplatin chemotherapy, mannitol showed no nephroprotective effect. After Al-Sarraf, there are four studies, i.e. by Santoso, Leu, Morgan and McKibbin et al. [33] The only randomized controlled trials, so far, held by Santoso et al., [17] reported that combination of hydration and mannitol resulted in significant decreased of 24-h creatine clearance rate (in ml/min) after chemotherapy compared to normal saline group (31 ± 2,7 vs 5,4 ± 5,1, *p* = 0.02) so the study was prematurely terminated due to the trend of worse outcomes with concomitant mannitol. Leu et al., [34], supported the result of Santoso study, revealed that combination of hydration and mannitol resulted in higher incidence of nephrotoxicity (9% vs 2%, *p* = 0.36). Meanwhile, Morgan et al., [35] reported that patients who did not receive mannitol had a higher risk of nephrotoxicity (OR 2.646; 95% CI = 1.008–6.944; *p* = 0,048). McKibbin et al., [33] supported the result of Morgan et al., reported that saline-mannitol combination resulted in lower nephrotoxicity (OR 0.16; 95% CI 0.04–0.65; *p* = 0,01). However, those three studies were retrospective, with the limitation of missing data analysis. Recent studied regarding the topic still had split conclusions. A study by Williams et al., [36] found elevation of serum creatinine is more common in patients without mannitol and therefore suggested that in Mannitol reduces nephrotoxicity in patients receiving ≥ 70 mg / m^2^ Cisplatin. In the other hand, a retrospective study by Begin et al. (2020) [37] stated that mannitol is more preferable in patients who receive < 75 mg / m^2^ in terms of nephroprotection and that there is no benefit of adding Mannitol when the dose of Cisplatin is ≥ 75 mg / m^2^. Meanwhile, a research by El Hamamsy et al., in (2017) [38] reported that when compared to hydration and acetazolamide, hydration and mannitol exhibits more frequent cisplatin-induced acute kidney injuries. However, Hamroun et al., [39] and Makimoto et al., P[40] stated that there’s still no compelling evidence of significance in the use of mannitol in regards of reducing nephrotoxicity.

In Cipto Mangunkusumo General Hospital (CMGH) and Mochtar Riady Comprehensive Cancer Center (MRCCC), the administration of mannitol was still debatable. Since there was no prevailing standard of prevention of cisplatin nephrotoxicity, the choice of mannitol administration was based on responsible physician clinical judgement. Therefore, we undertook this study to determine if there was any difference in the risk of AKI in solid organ cancer patients treated by high dose cisplatin (≥ 75 mg/m^2^), between patients receiving saline hydration alone compared to those receiving saline with addition of mannitol.

## Method

### Patient selection

This was an ambispective study (combination of prospective and retrospective methods) of solid organ cancer patients treated with high dose cisplatin (≥ 75 mg / m^2^) at the CMGH and MRCCC, Jakarta, approved by institutional review board. Data were collected from September 2017 until February 2018. On the prospective method, we took samples of patients who underwent high doses of cisplatin chemotherapy at the chemotherapy ward of CMGH since September 2017. On the retrospective method, the patient data were collected from the medical record of CMGH and MRCCC Siloam Hospitals. Patients were included if they were 18–60 years old, had a pathologically confirmed diagnosis of solid organ cancer, had an adequate baseline of glomerular filtration rate equal to or greater than 60 ml/minutes/1.73 m^2^, had a good performance status (Karnofsky score ≥ 80), received high dose cisplatin chemotherapy. The exclusion criteria were as follows: receive other potentially nephrotoxic drugs such as furosemide, non-steroidal anti-inflammatory drugs (NSAIDs), aminoglycosides, amphotericin B and cephalosporins or another chemotherapy agent (permetrexed, ifosfamide, gemcitabine, bevacizumab, cetuximab), had comorbidity such as malignant / uncontrolled hypertension with diastolic blood pressure ≥ 100 mmHg, congestive heart failure, any structural abnormalities of kidney (obstruction of the urinary tract, kidney cyst or kidney and urinary tract stones) diagnosed by radiology examination, suffered from any acute infection and was a pregnant women.

### Treatment schedule

The subjects were allocated into two groups: the group receiving the addition of mannitol to hydration prior to cisplatin chemotherapy, and the group receiving no mannitol. The administration of mannitol was based on clinical judgement of responsible physician, no intervention from researcher. Patient demographic information include age, sex, type of cancer, history of diabetes and history of hypertension, treatment data include mannitol use, chemotherapy regimen, cisplatin dose, history of previous cisplatin chemotherapy and cumulative cisplatin dose before current therapy, number of cycles of chemotherapy and concomitant radiotherapy. All subjects received the same saline-based hydration with 1-2L of 0.9% saline over 1–2 h and achieved euvolemic status before chemotherapy. All doses of cisplatin were diluted in 500 ml of 0.9% saline and infused over 2–3 h. Patients in the mannitol cohort received 20 g admixed in the 0.9% 100 ml saline prehydration. All patients received antiemetic premedication such as dexamethasone, a H_2_ receptor blocker and diphenhydramine.

### Assessment of outcome

The outcome of this study was any grade (grade 1 to 4) of AKI, was defined using the National Cancer Institute’s Common Terminology Criteria for Adverse Events (CTCAE v 4.0) grading scale for chemotherapy [41]. In this research, grade 1 AKI was used as the cutoff in determining whether the subject experience AKI or not. Grade 1 AKI according to the CTCAE was defined by an increase of more than 0.3 mg/dL or 1.5 times from baseline of serum creatinine. The evaluation of each patient is done twice: once before and once after a single cycle of cisplatin-based chemotherapy (where in this research, it could be the first cycle, the second, third or fourth cycle). For the latter, AKI is evaluated between 3 to 28 days after the respective cycle, before the next cycle [42].

### Statistical analysis

The collected data was processed using SPSS statistics program version 20.0. To evaluate the differences in the patient characteristics, the chi-square test was used. Bivariate analysis was performed to determine crude relative risk (RR) probability of post-chemotherapy AKI between mannitol group to non-mannitol group. Multivariate logistic regression models were created to assess the potential confounders and revealed adjusted RR. All analyzes used 5% significance limits.

## Results

### Patient characteristics

A total of 110 patients were included, 63 patients received addition of mannitol and 47 patients did not. The patient characteristics were listed in Table 1. The median age was 44.5 years old with an almost equal proportion of male and female (57.3% vs. 41.8%). More than 60% of patients diagnosed with head and neck cancer and treated with combination of cisplatin and 5-fluorouracil (44.5%). Most of patient was receiving first cycle of cisplatin chemotherapy, while some are undergoing their second, third and fourth cycle. Most patients received cisplatin dosage of 100 mg / m^2^ (67.3%). Greater than 95% of patients had never previous cisplatin chemotherapy series, so that cumulative dose received before current chemotherapy was lower than 100 mg/m^2^. Regarding comorbidities, only a small proportion of subjects were diagnosed with Diabetes Mellitus (2.7%) and hypertension (7.3%). For concomitant radiotherapy, most subjects (83.5%) were not given concomitant radiotherapy. All of patients were on good performance status before chemotherapy.

**Table 1:** Patient Demographics and Characteristics

	**Mannitol (*n* = 63)**	**Saline only (*n* = 47)**
**Sex**		
**Male,** n (%)	40 (63.5)	24 (51.1)
**Female,** n (%)	23 (36.5)	23 (48.9)
**Age,** median 44.5 yo		
≥ 40 years old (*n* = 71; 64.5)	32 (51.6)	39 (81.2)
< 40 years old (*n* = 39; 35.5)	31 (48.4)	8 (18.8)
**Type of cancer**		
Nasopharynx (*n* = 61; 55.5%)	41 (66.1)	20 (41.7)
Head and neck cancer other than nasopharyngeal (*n* = 14; 12.7%)	8 (12.9)	6 (12.5)
Osteosarcoma (*n* = 11; 10%)	8 (12.9)	3 (6.2)
Breast (*n* = 3; 2.7%)	0 (0)	3 (6.2)
Ovary (*n* = 4; 3.6%)	0 (0)	4 (8.5)
Others (*n* = 17; 15.5%)	5 8.0)	12 (25.1)
**Anticancer drugs**		
5FU (*n* = 49; 44.5%)	40 (64.5)	9 (18.8)
Docetaxel (*n* = 20; 18.2%)	10 (16.1)	9 (18.8)
Paclitaxel (*n* = 10; 9.1%)	2 (3.2)	8 (16.7)
Doxorubicin (*n* = 9; 8.2%)	7 (11.3)	3 (6.2)
Nimotuzumab (*n* = 7; 6.45%)	0 (0)	7 (14.6)
Etoposide (*n* = 5; 4.5%)	1 (1.6)	4 (8.3)
Others (*n* = 10; 9%)	2 (3.2)	8 (16.7)
**Dose of Cisplatin (mg/m**^**2**^**)**		
100 (*n* = 74; 67.3%)	53(84.1)	21(44.7)
80 (*n* = 7; 6.4%)	2(3.2)	5(10.6)
75 (*n* = 29; 6.4)	8(12.7)	21 (44.7)
**Cumulative dose of cisplatin before the current chemotherapy (mg/m**^**2**^**)**		
≤ 100, n (%)	52 (83.9)	38 (79.2)
101–200, n (%)	7 (11.3)	9 (18.8)
201–300, n (%)	2 (3.2)	1 (2.1)
> 300, n (%)	1 (1.6)	0
**Chemotherapy Cycle**		
1st (*n* = 69, 62.7%)	34 (54.0)	35 (74.5)
2nd (*n* = 22, 20%)	17 (27.0)	5 (10.6)
3rd (*n* = 3, 14%)	8 (12.7)	6 (12.8)
4th (*n* = 5, 4.5%)	4 (6.3)	1 (2.1)
**Diabetes Mellitus** (*n* = 3 [2.7])	0 (0)	3 (6.4)
**Hypertension** (*n* = 8 [7.3])	4 (6.3)	4 (8.5)
**Concomitant Radiotherapy**		
No (*n* = 91 [83.5])	55 (60.4)	36 (39.6)

*5FU* 5-fluorouracil

In this study, both groups received > 3.000 ml of hydration in 24 h pre and post chemotherapy, with an adequate urine production for 6 h post-chemotherapy of (mean value of 2.58 ± 1.01 ml / kgBW / hour). All subjects had good pre-chemotherapy renal function, i.e. median ureum 24 mg/dL, creatinine 0.8 mg /dL and glomerular filtration rate (CKD-EPI) was 103.85 ml / minute / 1.73 m2. No subjects had creatinine > 1.5 mg / dL pre-chemotherapy. All of subjects had a good median potassium value of 4.07 mEq / L with similar results on both groups. The clinical characteristics were listed in Table 2.

**Table 2:** Clinical Characteristics of Subjects

	**Before Chemotherapy**		**After Chemotherapy**	
	**Mannitol**	**No Mannitol**	**Mannitol**	**No Mannitol**
**Ureum**, median (min–max)	22 (4–68)	24 (10–47)	28.5 (8–76)	27 (13–81)
**Creatinine**, median (min–max) mg/dL	0.8 (0.3–1.2)	0.8 (0.29–1.4)	1.0 (0.4–2.2)	0.9 (0.41–2.3)
< 1, n (%)	55 (88.7)	43 (89.6)	37 (59.7)	35 (72.9)
1.1–1.5 n (%)	7 (11.3)	5 (10.4)	19 (30.6)	11 (22.9)
> 1.5 n (%)	0	0	6 (9.7)	2 (4.2)
**eGFR**, median (min–max) **ml/minute/1.73m**^**2**^	106.5 (62.7–169.8)	102 (60–253.4)	90.11 ± 28.67	90.48 ± 35.58
** < 60**, n (%)	0	0	11 (17.7)	5 (10.4)
**60–90,** n (%)	14 (22.6)	14 (29.2)	18 (29)	22 (45.8)
** > 90,** n (%)	48 (77.4)	34 (70.8)	33 (53.2)	21 (43.8)
**Potassium**, median (min–max)	4.14 (3.03–5.46)	3.9 (2.8–5.01)	3.845 (2.8–5.37)	4.0 (2.3–5.1)
**Pre-chemotherapy hydration**, mean ± SD	4270.95 ± 740.11	3150.63 ± 656.28	4215.32 ± 759.47	3322.34 ± 780.87

*eGFR* estimated glomerular filtration rate

### Incidence and outcome of AKI

**Fig. 1:**
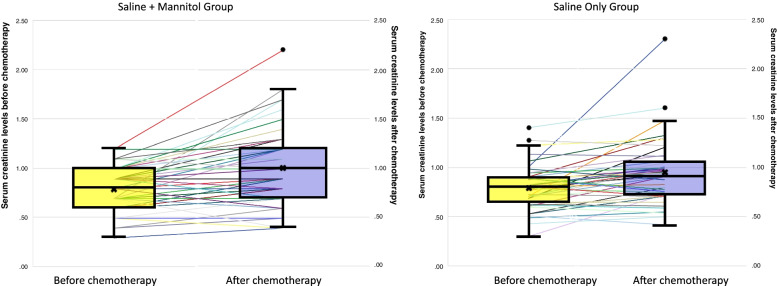
The comparison of creatinine levels before and after chemotherapy in the saline + mannitol and the saline only group

The incidence of AKI was observed in 14 patients (22.6%) in mannitol group versus 5 patients (10.4%) in saline only group, however it was not statistically different (*p* value = 0.076; RR 2.168; 95% CI 0.839–5.6). All subjects experience an increase of serum creatinine after chemotherapy. The decrease in renal function was more noticeable in mannitol group than no mannitol group. The increase of serum creatinine was 29% in mannitol vs. 16.7% in no mannitol group. The increase of serum creatinine from less than 1.0 mg/dL to 1.1–1.5 mg / dL was found to be greater in the mannitol group than saline only group (30.6% vs 22.9%). In addition, the increase in creatinine to more than 1.5 mg / dL were found to be higher in the group receiving mannitol than without mannitol (9.7% vs 4.2%). Similarly, a decrease in GFR from more than 90 ml/min/1.73 m^2^ to less than 60 ml/min/1.73 m^2^, was greater in the mannitol group than without mannitol (17.7% vs. 10.4%). The pre-post chemotherapy comparison of renal function is detailed in Table 3 and shown in Fig. 1.

**Table 3:** The mean decline of pre-and post-chemotherapy renal function in the mannitol and no mannitol groups

	**Manitol (IQR)**	**Saline only (IQR)**	***p***
Median creatinine difference	0.20 (0—0,30)	0.12 (-0,015—0,215)	0.166
Median eGFR difference	15.10 (0—32.63)	11.75(-2.78—27.40)	0.349

*eGFR* estimated glomerular filtration rate, *IQR* interquartile range

### Potential risk factors for developing AKI

We conducted bivariate analysis to analyze the potential confounding factor for such variables: age, sex, type of cancer, chemotherapy regimen, cisplatin dose, chemotherapy cycle, comorbidity of diabetes and hypertension and the administration of concomitant radiotherapy (Table 4). The variables having *p* values < 0.25 in bivariate analysis were included in the multivariate analysis. Based on bivariate analysis, the variables included in the multivariate analysis were age and chemotherapy regimen. Multivariate analysis and changes of crude RR (relative risk) to be adjusted RR between mannitol and post-chemotherapy AKI incidence by controlling the potential confounding factors were listed in Table 5.

**Table 4:** The relationship between potential confounding factors and post-cisplatin acute kidney injury

Variables	Post chemotherapy AKI (%)		*p*
	**Yes**	**No**	
**Age**			
< 40 years old	10.3	89.7	0.149
≥ 40 years old	21.1	78.9	
**Sex**			
Male	18.5	81.5	0.692
Female	15.6	84.4	
**Type of Cancer**			
Nasopharyngeal	18.0	82.0	0.467
Head and neck other than nasopharyngeal	7.1	92.9	
Osteosarcoma	9.1	90.9	
Others	25	75	
**Regimen of Chemotherapy**			
5FU	26.5	73.5	0.146
Docetaxel	10.5	89.5	
Paclitaxel	10.0	90.0	
Doxorubicin	0	100	
Others	13.6	86.4	
**Dose of Cisplatin (**mg/m^2^)			
100	10.3	89.7	0.525
80	0	100	
75	21.6	78.4	
**Cycle of Chemotherapy**			
1st	16.2	83.8	1.00
2 nd	21.7	78.3	
3 rd	20.0	80.0	
4 th	0	100	
**Diabetes Mellitus**	0	100	1.00
No DM	17.8	82.2	
**Hypertension**	36.4	63.6	0.942
No Hypertension	15.2	84.8	
***Concomitant Radiotherapy***			
Yes	11.1	89.8	0.734
No	18.5	81.5

**Table 5:** Crude RR and Adjusted RR with 95% CI of mannitol on post-chemotherapy AKI with the addition of potential confounding factors gradually

Variables	RR (CI 95%)	*P* value	RR value changes with confounder
***Crude RR***	2.168 (0.839–5.6)	0.094	
***Adjusted RR***			
+ Chemotherapy regimen 5 fluorouracil	2.190 (0.555–8.632)	0.448	(2.190–2.168)/2.190 × 100% = 1%
+ Age ≥ 40 years old	2.852 (0.68–11.96)	**0.152**	(2.852–2.190)/2.190 × 100% = 23%

*RR* Relative Risk

On multivariate analysis by controlling age and chemotherapy regimen, the risk of post cisplatin AKI was greater in saline with addition of mannitol than saline only (adjusted RR was 2.852; 95% CI 0.68–11.96).

## Discussion

Our data suggests that the addition of mannitol to saline hydration did not aid in decreasing the risk of cisplatin-induced AKI compared when compared to hydration using saline solution (*p* value = 0.076; RR 2.168; 95% CI 0.839–5.6). Age ≥ 40 years old was concluded to be a confounding factor and may exhibit higher risk of AKI (adjusted RR 2.852; 95% CI 0.68—11.96).

Our study is in line with a recent study by Begin et al.[37] which stated that there was no difference in AKI incidence between subject which was given mannitol in addition to hydration and hydration alone (HR 1.17 [0.75–1.82]) after administration of cisplatin ≥ 75 mg/m^2^. There were different hydration protocols between this study and our study. They used 3 L before and 1 L of hydration after cisplatin chemotherapy, whereas ours used 1 L before chemotherapy [37]. Our findings were also supported by the study by Leu et al. [34], who reported the tendency of greater risk of nephrotoxicity in saline and mannitol group versus saline only (the decrease of creatinine clearance was 38. 9 ml/min vs. 33.9 ml/min, *p* = 0.09) [34].

Other studies found that addition of mannitol increase the risk of AKI. One was the study by Santoso, et al. [17] in the United States. Santoso et al., conducted a randomized controlled clinical trial found that decreased renal function, in this study assessed by 24 h creatinine clearance, occurred more heavily in the group given the combination of hydration and mannitol than hydration alone (31 ml/min vs 5.4 ml/min, *p* value = 0.04)[17]. The study was discontinued prematurely because of higher tendency of nephrotoxicity in mannitol group, so that the expected sample size was not achieved (there were only 49 subjects). The discontinuation of study showed that nephrotoxicity potency of mannitol which supports our findings.

Our results differ from those of Hayes et al. [16], Morgan et al. [35], and McKibbin et al. [33] which found that the addition of mannitol decrease the risk of AKI. However, there are notable limitations of those studies. The study by Hayes et al., [16] was non-comparative trial (no comparison data with patients receiving saline only), so it was difficult to analyze whether the nephroprotective outcome came from mannitol or adequate hydration only. The study by Morgan et al., which reported the higher risk of nephrotoxicity from the group without mannitol (OR 2.646 (95% CI 1.008–6.944; *p* = 0.048), was a retrospective study and had small sample size (only 47 patients received high dose cisplatin). The study by McKibbin et al., [33] which support nephroprotective effect of mannitol after multivariate analysis (odds ratio of third grade nephrotoxicity in mannitol group was 0.16; 95% CI 0.04–0.65, *p* value = 0.01) had a limitation in the analysis of concomitant use of nephrotoxic substance due to missing data because of the retrospective nature of study.

The underlying mechanisms of nephrotoxicity of mannitol was through the osmotic effect of mannitol which inhibits the reabsorption of water in the proximal tubule, resulted in urinary dilution and an increased diuresis. In one side, this effect decreased the contact time of cisplatin with renal tubular cells and increased the clearance of necrotic cell debris at renal tubules after injured by cisplatin. However, this mechanism seemed to have nephrotoxic potential, which was related with hemodynamic changes in the kidney. Mannitol triggered a marked decrease in the reabsorption of water and salt along the renal tubules, resulted in increased flow of water and salts from the proximal tubules, followed by increased sodium reabsorption in loop of Henle, distal tubules and collecting ducts. Increased excretion of urine solutes induced by the mannitol osmotic diuretic effect lead to increased tubuloglomerular feedback which stimulate afferent arteriolar vasoconstriction, hence resulted in decrease of glomerular filtration rate [43, 44]. Besides that, mannitol would lead the osmotic nephrosis effect on renal tubules. Histologically, tubular cells with toxic effects of mannitol appeared to contain vacuoles resulting in edema, called osmotic nephrosis. Pathophysiologically, the mechanism was through the pinocytosis effect of mannitol into the proximal tubular cell at high osmolality which then causes tubular cell vacuolization. These vacuoles would become fused and develop an edematous cell, resulting an obstruction of renal tubules [45], then led to a decline in glomerular flow and AKI*.* Meta-analysis of Bo Yang et al. [31] in 626 subjects revealed that intravascular mannitol administration did not provide additional benefit than adequate hydration alone in patients at risk of AKI, however in contrast-induced nephropathy, the effect was even detrimental [31].

We determined age as a confounding variable. Decreased renal function with increasing age was associated with decreased plasma flow velocity in glomerular capillaries and glomerular capillary ultrafiltration coefficient. In addition, there were hemodynamic changes associated with structural changes such as decreased renal mass, increased sclerotic glomeruli and tubulointerstitial fibrosis [46]. The Davies and Shock study of inulin clearance reported a glomerular filtration rate decrease of 8 ml / min / 1.73 m^2^ in each year from the age of 40 years old [47]. The increasing trend of incidence of AKI with age was consistent with previous research results from Prasaja et al., [48] which reported that over 50 years of age have a higher risk of nephrotoxicity after four cycles of chemotherapy (OR 3.433; 95% CI 1.363–8.645). The study from Perazella et al. [49], Caglar et al. [22], and de Jongh et al. [50], revealed same result, that advancing age was one of the factors that increased the risk of nephrotoxicity [49].

Our study did not find the desired nephroprotective effect off adding mannitol to saline hydration. We also included different types of cancer type, our study only included subjects who received high doses of chemotherapy and excluded subjects who received nephrotoxic drugs simultaneously, two important things that became a limitation in previous studies. For the outcome of renal function, we used serum creatinine parameters, as recommended by the National Cancer Institute's Common Terminology Criteria for Adverse Events (CTCAE v 4.0) grading scale for chemotherapy to minimize bias due to other measures [41].

### Study limitation

There are several other things to consider when evaluating these results. Most notably, because of cohort nature of this study, we did not randomize patients to receive or not receive mannitol, rather we submitted a decision based on clinical judgement of responsible physician. The dose and cycle cisplatin regimen also vary from subject to subject. For future studies we suggest performing a randomized-controlled trial with more homogenous subject in terms of the dose and cycle of the chemotherapy regimen.

Besides that, our study did not analyze fluid intake at home and excess fluid loss caused by vomiting as a side effect of cisplatin chemotherapy. However, all our subjects had the same approach of post chemotherapy nausea and vomiting; the medication for nausea and vomiting prophylaxis was given to all subjects. There is a need for future prospective study where fluid intake and water balance are strictly controlled to determine better the magnitude of risk from mannitol.

We hope the results of our study might become a consideration regarding the policy of addition of mannitol to hydration in cisplatin chemotherapy. This might have an added benefit in the cost-effectiveness of chemotherapy if administration of mannitol is no longer routinely given in high-dose cisplatin chemotherapy nowadays.

## Conclusion

In our study, the addition of mannitol to hydration did not reduce the risk of cisplatin induced AKI as compared with saline hydration only. It was also found that risk for AKI were higher in population ≥ 40 years old.

## Data Availability

The authors confirm that the data supporting the findings of this study are available within the article and its supplementary material. Raw data that support the findings of the study are available from the corresponding author, upon reasonable request.

## Abbreviations

AKI: Acute kidney injury
CI: Confidence interval
CMGH: Dr. Cipto Mangunkusumo General Hospital
CTCAE: Common Terminology Criteria for Adverse Events
DM: Diabetes Mellitus
5FU: Fluorouracil
GFR: Glomerular filtration rate
HR: Hazard ratio
MRCCC: Mochtar Riady Comprehensive Cancer Center
NSAID: Non-steroid anti-inflammatory drugs
OR: Odds ratio
RR: Relative risk
